# [η^5^-1,3-Bis(trimethyl­sil­yl)cyclo­penta­dien­yl]dichlorido[η^5^-(trimethyl­sil­yl)cyclo­penta­dien­yl]titanium(IV)

**DOI:** 10.1107/S1600536811046228

**Published:** 2011-11-05

**Authors:** Franc Perdih

**Affiliations:** a Faculty of Chemistry and Chemical Technology, University of Ljubljana, Aškerčeva 5, PO Box 537, SI-1000 Ljubljana, Slovenia, and CO EN–FIST, Dunajska 156, SI-1000 Ljubljana, Slovenia

## Abstract

In the title compound, [Ti(C_8_H_13_Si)(C_11_H_21_Si_2_)Cl_2_], the Ti^IV^ atom is bonded to two Cl atoms, one 1,3-bis­(trimethyl­sil­yl)cyclo­penta­dienyl (Si_2_Cp) and one (trimethyl­sil­yl)cyclo­penta­dienyl ring (SiCp). The Si_2_Cp centroid–titanium distance is 2.0763 (10) Å and the SiCp centroid–titanium distance is 2.0793 (10) Å. The angle subtended at the Ti atom by the centroids of both cyclo­penta­dienyl rings is 131.22 (4)° and the Cl—Ti—Cl angle is 94.14 (2)°.

## Related literature

For background to metallocene catalysts, see: Kaminsky *et al.* (2006 ▶); Erker *et al.* (2006 ▶); Alt *et al.* (2006 ▶); Zhu *et al.* (2010 ▶); Luo *et al.* (2011 ▶); Winter *et al.* (1992 ▶); Möhring & Coville (2006 ▶). For related structures, see: Klouras & Nastopoulos (1991 ▶); Clearfield *et al.* (1975 ▶); McKenzie *et al.* (1975 ▶); Winter *et al.* (1992 ▶). For synthetic procedures, see: Winter *et al.* (1992 ▶).
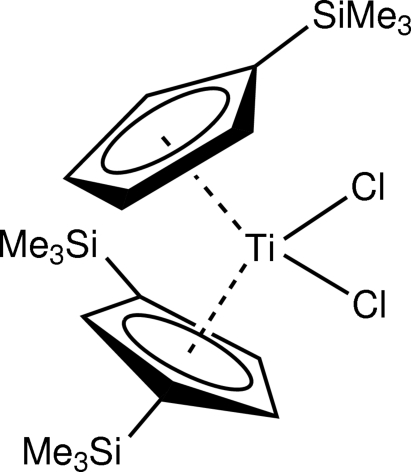


## Experimental

### 

#### Crystal data

[Ti(C_8_H_13_Si)(C_11_H_21_Si_2_)Cl_2_]
*M* *_r_* = 465.53
Monoclinic, 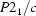

*a* = 10.2588 (2) Å
*b* = 18.6417 (4) Å
*c* = 13.3061 (2) Å
β = 105.2380 (12)°
*V* = 2455.21 (8) Å^3^
*Z* = 4
Mo *K*α radiation
μ = 0.72 mm^−1^
*T* = 150 K
0.2 × 0.2 × 0.2 mm

#### Data collection

Nonius KappaCCD area-detector diffractometer
Absorption correction: multi-scan (*SCALEPACK*; Otwinowski & Minor, 1997 ▶) *T*_min_ = 0.870, *T*_max_ = 0.870
10555 measured reflections
5586 independent reflections
4437 reflections with *I* > 2σ(*I*)
*R*_int_ = 0.024

#### Refinement

*R*[*F* ^2^ > 2σ(*F* ^2^)] = 0.037
*wR*(*F* ^2^) = 0.091
*S* = 1.03
5586 reflections
235 parameters
H-atom parameters constrained
Δρ_max_ = 0.34 e Å^−3^
Δρ_min_ = −0.37 e Å^−3^

### 

Data collection: *COLLECT* (Nonius, 1998 ▶); cell refinement: *DENZO-SMN* (Otwinowski & Minor, 1997 ▶); data reduction: *DENZO-SMN*; program(s) used to solve structure: *SHELXS97* (Sheldrick, 2008 ▶); program(s) used to refine structure: *SHELXL97* (Sheldrick, 2008 ▶); molecular graphics: *ORTEP-3* (Farrugia, 1997 ▶); software used to prepare material for publication: *WinGX* (Farrugia, 1999 ▶) and *publCIF* (Westrip, 2010 ▶).

## Supplementary Material

Crystal structure: contains datablock(s) I, global. DOI: 10.1107/S1600536811046228/fj2469sup1.cif

Structure factors: contains datablock(s) I. DOI: 10.1107/S1600536811046228/fj2469Isup2.hkl

Additional supplementary materials:  crystallographic information; 3D view; checkCIF report

## Figures and Tables

**Fig. 1.**
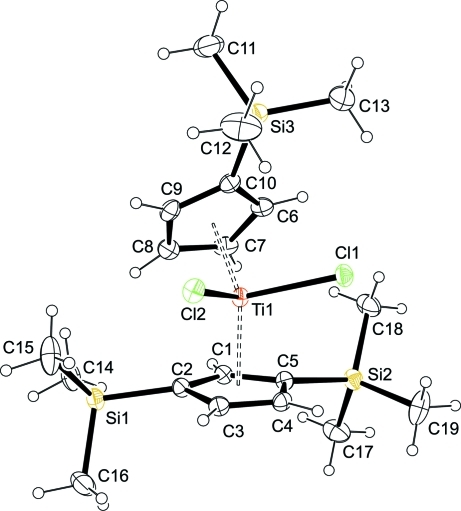
The molecular structure of the title compound showing the numbering scheme and displacement ellipsoids drawn at the 30% probability level.

**Fig. 2.**
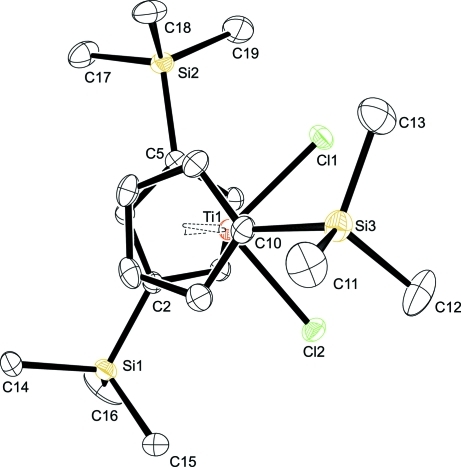
Top view of the molecular structure of the title compound with displacement ellipsoids drawn at the 30% probability level. H atoms are omitted for clarity.

## supplementary crystallographic information

### Comment

Metallocene-based catalysts for homogeneous polymerization have developed markedly over the last three decades (Kaminsky *et al.*, 2006; Erker *et al.*, 2006; Alt *et al.*, 2006; for recent related XRD studies, see: Zhu *et al.*, 2010; Luo *et al.*, 2011). Metallocenes bearing bulky substituents often exhibit properties very different from those of the corresponding unsubstituted analogues. Although pentamethylcyclopentadienyl ligand is one among most often used, other bulky cyclopentadienyl ligands have also been employed (Winter *et al.*, 1992; Möhring & Coville, 2006).

In the title compound [Ti(C_11_H_21_Si_2_)(C_8_H_13_Si)Cl_2_] or [Ti{C_5_H_3_(SiMe_3_)_2_}{C_5_H_4_(SiMe_3_)}Cl_2_] titanium atom is bonded to two chlorine atoms, one 1,3-bis(trimethylsilyl)cyclopentadienyl and one (trimethylsilyl)cyclopentadienyl ring (Figs. 1–2). The Si_2_Cp centroid–titanium distance is 2.0763 (10) Å and the SiCp centroid–titanium distance is 2.0793 (10) Å. The *Cg*1–Ti–*Cg*2 angle between the cyclopentadienyl ligands of 131.22 (4)° is similar to the value of 131.02° in [Ti{C_5_H_4_(SiMe_3_)}_2_Cl_2_] (Klouras & Nastopoulos, 1991) but slightly larger than the value of 130.89° and 131.04° in the C_5_H_5_ case (Clearfield *et al.*, 1975) and smaller than the value of 137.4° in the C_5_Me_5_ case (McKenzie *et al.*, 1975).

One measure of steric interactions in substituted cyclopentadienyl compounds is the degree to which the cyclopenadienyl substituents are bent out of the plane of the cyclopentadienyl ligand. The angle between the Si–C(Cp) bond and the plane of the Cp ring for Si1, Si2 and Si3 are 9.10 (11)°, 7.85 (11)° and 9.25 (11)°, respectively. For comparison, this values in related [Ti{C_5_H_3_(SiMe_3_)_2_}_2_F_2_] are from 0.6° to 6.6°, while in [Ti{C_5_H_2_(SiMe_3_)_3_}{C_5_H_4_(SiMe_3_)}F_2_] are from 1.0° to 6.9° (Winter *et al.*, 1992).

In the crystal structure there are no hydrogen bonds or π–π interactions.

### Experimental

1,1',3-Tris(trimethylsilyl)titanocene dichloride was prepared according to the published procedure (Winter *et al.*, 1992). Red crystals suitable for single-crystal *X*– ray diffraction were grown at -5°C from hexane.

### Refinement

All H atoms were initially located in a difference Fourier maps and were subsequently treated as riding atoms in geometrically idealized positions, with C—H = 0.95 (aromatic) or 0.98 Å (CH_3_), and with *U*_iso_(H) = *kU*_eq_(C), where *k* = 1.5 for methyl groups, which were permitted to rotate but not to tilt, and 1.2 for all other H atoms.

### Crystal data

**Table:**

[Ti(C_8_H_13_Si)(C_11_H_21_Si_2_)Cl_2_]	*F*(000) = 984
*M**_r_* = 465.53	*D*_x_ = 1.259 Mg m^−^^3^
Monoclinic, *P*2_1_/*c*	Mo *K*α radiation, λ = 0.71073 Å
Hall symbol: -P 2ybc	Cell parameters from 5741 reflections
*a* = 10.2588 (2) Å	θ = 2.6–27.5°
*b* = 18.6417 (4) Å	µ = 0.72 mm^−^^1^
*c* = 13.3061 (2) Å	*T* = 150 K
β = 105.2380 (12)°	Cube, red
*V* = 2455.21 (8) Å^3^	0.2 × 0.2 × 0.2 mm
*Z* = 4	

### Data collection

**Table:**

Nonius KappaCCD area-detector diffractometer	5586 independent reflections
graphite	4437 reflections with *I* > 2σ(*I*)
Detector resolution: 0.055 pixels mm^-1^	*R*_int_ = 0.024
ω scans	θ_max_ = 27.4°, θ_min_ = 3.6°
Absorption correction: multi-scan (*SCALEPACK*; Otwinowski & Minor, 1997)	*h* = −13→13
*T*_min_ = 0.870, *T*_max_ = 0.870	*k* = −24→24
10555 measured reflections	*l* = −17→17

### Refinement

**Table:**

Refinement on *F*^2^	Primary atom site location: structure-invariant direct methods
Least-squares matrix: full	Secondary atom site location: difference Fourier map
*R*[*F*^2^ > 2σ(*F*^2^)] = 0.037	Hydrogen site location: inferred from neighbouring sites
*wR*(*F*^2^) = 0.091	H-atom parameters constrained
*S* = 1.03	*w* = 1/[σ^2^(*F*_o_^2^) + (0.0373*P*)^2^ + 1.6583*P*] where *P* = (*F*_o_^2^ + 2*F*_c_^2^)/3
5586 reflections	(Δ/σ)_max_ = 0.001
235 parameters	Δρ_max_ = 0.34 e Å^−^^3^
0 restraints	Δρ_min_ = −0.37 e Å^−^^3^

### Special details

**Table:**

Experimental. 224 frames in 6 sets of ω scans. Rotation/frame = 1.8 °. Crystal-detector distance = 31.0 mm. Measuring time = 190 s/°.
Geometry. All e.s.d.'s (except the e.s.d. in the dihedral angle between two l.s. planes) are estimated using the full covariance matrix. The cell e.s.d.'s are taken into account individually in the estimation of e.s.d.'s in distances, angles and torsion angles; correlations between e.s.d.'s in cell parameters are only used when they are defined by crystal symmetry. An approximate (isotropic) treatment of cell e.s.d.'s is used for estimating e.s.d.'s involving l.s. planes.
Refinement. Refinement of *F*^2^ against ALL reflections. The weighted *R*-factor *wR* and goodness of fit *S* are based on *F*^2^, conventional *R*-factors *R* are based on *F*, with *F* set to zero for negative *F*^2^. The threshold expression of *F*^2^ > σ(*F*^2^) is used only for calculating *R*-factors(gt) *etc*. and is not relevant to the choice of reflections for refinement. *R*-factors based on *F*^2^ are statistically about twice as large as those based on *F*, and *R*- factors based on ALL data will be even larger.

### Fractional atomic coordinates and isotropic or equivalent isotropic displacement parameters (Å^2^)

**Table:**

	*x*	*y*	*z*	*U*_iso_*/*U*_eq_	
Ti1	0.92494 (3)	0.234506 (19)	0.42358 (3)	0.02364 (10)	
Cl1	0.71504 (5)	0.24806 (3)	0.46198 (4)	0.03426 (13)	
Cl2	1.01487 (6)	0.15818 (3)	0.56780 (4)	0.03435 (13)	
Si1	1.30658 (6)	0.27181 (4)	0.45355 (5)	0.03322 (15)	
Si2	0.78071 (6)	0.42245 (3)	0.33826 (5)	0.03503 (15)	
Si3	0.71083 (7)	0.05471 (3)	0.35754 (5)	0.03846 (16)	
C1	1.0327 (2)	0.33664 (11)	0.37062 (15)	0.0275 (4)	
H1	1.0392	0.3416	0.301	0.033*	
C2	1.1309 (2)	0.30217 (11)	0.45156 (15)	0.0284 (4)	
C3	1.0828 (2)	0.31075 (12)	0.54242 (15)	0.0300 (4)	
H3	1.1285	0.2947	0.6103	0.036*	
C4	0.9591 (2)	0.34617 (11)	0.51647 (15)	0.0291 (4)	
H4	0.9067	0.3576	0.5635	0.035*	
C5	0.9235 (2)	0.36265 (11)	0.40747 (15)	0.0280 (4)	
C6	0.7751 (2)	0.18915 (12)	0.26451 (15)	0.0334 (5)	
H6	0.6838	0.2046	0.2424	0.04*	
C7	0.8836 (2)	0.22377 (13)	0.24119 (16)	0.0362 (5)	
H7	0.8795	0.2663	0.2012	0.043*	
C8	1.0014 (2)	0.18388 (13)	0.28814 (16)	0.0368 (5)	
H8	1.0909	0.195	0.2857	0.044*	
C9	0.9620 (2)	0.12503 (12)	0.33887 (16)	0.0350 (5)	
H9	1.0211	0.0891	0.376	0.042*	
C10	0.8207 (2)	0.12731 (11)	0.32631 (15)	0.0310 (4)	
C11	0.7207 (3)	−0.02067 (16)	0.2689 (3)	0.0641 (8)	
H11A	0.6925	−0.004	0.1965	0.096*	
H11B	0.661	−0.0595	0.2788	0.096*	
H11C	0.8139	−0.0383	0.2844	0.096*	
C12	0.7701 (4)	0.02490 (18)	0.4949 (2)	0.0724 (10)	
H12A	0.7092	−0.0122	0.5085	0.109*	
H12B	0.7708	0.0658	0.5413	0.109*	
H12C	0.8616	0.0053	0.5076	0.109*	
C13	0.5332 (3)	0.08662 (16)	0.3299 (3)	0.0618 (8)	
H13A	0.4986	0.0961	0.2551	0.093*	
H13B	0.5298	0.1308	0.369	0.093*	
H13C	0.4775	0.0498	0.3509	0.093*	
C14	1.3327 (2)	0.27575 (16)	0.32045 (19)	0.0469 (6)	
H14A	1.2867	0.318	0.2839	0.07*	
H14B	1.2956	0.2324	0.2818	0.07*	
H14C	1.4296	0.2789	0.3256	0.07*	
C15	1.3414 (3)	0.18025 (18)	0.5086 (3)	0.0638 (9)	
H15A	1.2916	0.1451	0.4582	0.096*	
H15B	1.3126	0.1769	0.5731	0.096*	
H15C	1.4385	0.1704	0.5237	0.096*	
C16	1.4201 (3)	0.3360 (2)	0.5413 (3)	0.0824 (12)	
H16A	1.5141	0.3206	0.5519	0.124*	
H16B	1.3978	0.3374	0.6085	0.124*	
H16C	1.4084	0.3839	0.5099	0.124*	
C17	0.8614 (3)	0.49936 (15)	0.2878 (2)	0.0541 (7)	
H17A	0.9104	0.4816	0.2387	0.081*	
H17B	0.9246	0.5236	0.346	0.081*	
H17C	0.7916	0.5333	0.2521	0.081*	
C18	0.6585 (3)	0.37676 (14)	0.2286 (2)	0.0508 (7)	
H18A	0.6149	0.3372	0.2558	0.076*	
H18B	0.7066	0.3579	0.1796	0.076*	
H18C	0.5898	0.4112	0.1926	0.076*	
C19	0.6945 (3)	0.45443 (17)	0.4363 (3)	0.0675 (9)	
H19A	0.6168	0.4842	0.4019	0.101*	
H19B	0.7577	0.4828	0.4894	0.101*	
H19C	0.6634	0.4131	0.4692	0.101*	

### Atomic displacement parameters (Å^2^)

**Table:**

	*U*^11^	*U*^22^	*U*^33^	*U*^12^	*U*^13^	*U*^23^
Ti1	0.02510 (18)	0.02871 (19)	0.01821 (16)	0.00288 (14)	0.00763 (13)	0.00125 (14)
Cl1	0.0271 (2)	0.0417 (3)	0.0371 (3)	0.0018 (2)	0.0138 (2)	−0.0020 (2)
Cl2	0.0404 (3)	0.0375 (3)	0.0245 (2)	0.0065 (2)	0.0073 (2)	0.0061 (2)
Si1	0.0254 (3)	0.0444 (4)	0.0301 (3)	0.0038 (3)	0.0077 (2)	−0.0004 (3)
Si2	0.0319 (3)	0.0316 (3)	0.0409 (3)	0.0044 (2)	0.0085 (3)	0.0080 (3)
Si3	0.0456 (4)	0.0308 (3)	0.0402 (3)	−0.0033 (3)	0.0135 (3)	0.0001 (3)
C1	0.0278 (10)	0.0312 (11)	0.0243 (9)	−0.0008 (8)	0.0083 (8)	0.0015 (8)
C2	0.0268 (10)	0.0334 (11)	0.0255 (9)	−0.0005 (8)	0.0078 (8)	−0.0007 (8)
C3	0.0326 (11)	0.0353 (11)	0.0212 (9)	−0.0002 (9)	0.0056 (8)	−0.0022 (8)
C4	0.0334 (11)	0.0310 (11)	0.0254 (10)	0.0000 (9)	0.0120 (8)	−0.0029 (8)
C5	0.0275 (10)	0.0290 (10)	0.0273 (10)	0.0010 (8)	0.0070 (8)	0.0021 (8)
C6	0.0394 (12)	0.0343 (12)	0.0230 (10)	−0.0023 (9)	0.0020 (9)	−0.0017 (9)
C7	0.0526 (14)	0.0381 (12)	0.0180 (9)	−0.0084 (10)	0.0092 (9)	−0.0025 (9)
C8	0.0384 (12)	0.0495 (14)	0.0269 (10)	−0.0067 (10)	0.0164 (9)	−0.0117 (10)
C9	0.0432 (13)	0.0355 (12)	0.0282 (10)	0.0055 (10)	0.0129 (10)	−0.0067 (9)
C10	0.0394 (12)	0.0292 (11)	0.0252 (10)	0.0007 (9)	0.0102 (9)	−0.0021 (8)
C11	0.076 (2)	0.0430 (16)	0.077 (2)	−0.0130 (14)	0.0278 (17)	−0.0178 (14)
C12	0.098 (3)	0.061 (2)	0.0527 (17)	−0.0260 (18)	0.0103 (17)	0.0199 (15)
C13	0.0491 (16)	0.0462 (16)	0.093 (2)	−0.0067 (13)	0.0235 (16)	−0.0016 (16)
C14	0.0336 (12)	0.0719 (18)	0.0394 (13)	0.0101 (12)	0.0168 (10)	0.0136 (12)
C15	0.0499 (16)	0.076 (2)	0.0739 (19)	0.0297 (15)	0.0312 (15)	0.0387 (17)
C16	0.0327 (14)	0.114 (3)	0.100 (3)	−0.0141 (17)	0.0159 (16)	−0.061 (2)
C17	0.0524 (16)	0.0399 (14)	0.0620 (17)	−0.0081 (12)	0.0010 (13)	0.0171 (13)
C18	0.0360 (13)	0.0431 (14)	0.0625 (17)	−0.0001 (11)	−0.0059 (12)	0.0117 (12)
C19	0.073 (2)	0.065 (2)	0.074 (2)	0.0377 (17)	0.0348 (17)	0.0128 (16)

### Geometric parameters (Å, °)

**Table:**

Ti1—C8	2.344 (2)	C6—H6	0.95
Ti1—Cl1	2.3537 (6)	C7—C8	1.416 (3)
Ti1—C7	2.360 (2)	C7—H7	0.95
Ti1—Cl2	2.3723 (6)	C8—C9	1.402 (3)
Ti1—C5	2.398 (2)	C8—H8	0.95
Ti1—C1	2.399 (2)	C9—C10	1.416 (3)
Ti1—C4	2.399 (2)	C9—H9	0.95
Ti1—C2	2.404 (2)	C11—H11A	0.98
Ti1—C3	2.408 (2)	C11—H11B	0.98
Ti1—C9	2.410 (2)	C11—H11C	0.98
Ti1—C6	2.418 (2)	C12—H12A	0.98
Ti1—C10	2.467 (2)	C12—H12B	0.98
Si1—C16	1.854 (3)	C12—H12C	0.98
Si1—C15	1.855 (3)	C13—H13A	0.98
Si1—C14	1.861 (2)	C13—H13B	0.98
Si1—C2	1.883 (2)	C13—H13C	0.98
Si2—C19	1.856 (3)	C14—H14A	0.98
Si2—C18	1.862 (3)	C14—H14B	0.98
Si2—C17	1.867 (3)	C14—H14C	0.98
Si2—C5	1.878 (2)	C15—H15A	0.98
Si3—C12	1.853 (3)	C15—H15B	0.98
Si3—C11	1.855 (3)	C15—H15C	0.98
Si3—C13	1.860 (3)	C16—H16A	0.98
Si3—C10	1.876 (2)	C16—H16B	0.98
C1—C2	1.420 (3)	C16—H16C	0.98
C1—C5	1.421 (3)	C17—H17A	0.98
C1—H1	0.95	C17—H17B	0.98
C2—C3	1.430 (3)	C17—H17C	0.98
C3—C4	1.391 (3)	C18—H18A	0.98
C3—H3	0.95	C18—H18B	0.98
C4—C5	1.433 (3)	C18—H18C	0.98
C4—H4	0.95	C19—H19A	0.98
C6—C7	1.391 (3)	C19—H19B	0.98
C6—C10	1.422 (3)	C19—H19C	0.98
			
C8—Ti1—Cl1	136.06 (6)	C4—C3—H3	125.1
C8—Ti1—C7	35.04 (8)	C2—C3—H3	125.1
Cl1—Ti1—C7	107.76 (6)	Ti1—C3—H3	121.1
C8—Ti1—Cl2	104.05 (6)	C3—C4—C5	109.03 (18)
Cl1—Ti1—Cl2	94.14 (2)	C3—C4—Ti1	73.52 (12)
C7—Ti1—Cl2	135.04 (6)	C5—C4—Ti1	72.58 (12)
C8—Ti1—C5	109.13 (8)	C3—C4—H4	125.5
Cl1—Ti1—C5	85.88 (5)	C5—C4—H4	125.5
C7—Ti1—C5	89.85 (7)	Ti1—C4—H4	120.1
Cl2—Ti1—C5	131.43 (5)	C1—C5—C4	105.19 (17)
C8—Ti1—C1	79.66 (8)	C1—C5—Si2	128.13 (15)
Cl1—Ti1—C1	119.82 (5)	C4—C5—Si2	125.58 (16)
C7—Ti1—C1	74.77 (7)	C1—C5—Ti1	72.79 (12)
Cl2—Ti1—C1	127.05 (5)	C4—C5—Ti1	72.67 (12)
C5—Ti1—C1	34.46 (7)	Si2—C5—Ti1	128.36 (10)
C8—Ti1—C4	135.41 (8)	C7—C6—C10	110.2 (2)
Cl1—Ti1—C4	79.24 (5)	C7—C6—Ti1	70.79 (12)
C7—Ti1—C4	124.46 (8)	C10—C6—Ti1	74.96 (12)
Cl2—Ti1—C4	97.50 (5)	C7—C6—H6	124.9
C5—Ti1—C4	34.75 (7)	C10—C6—H6	124.9
C1—Ti1—C4	56.39 (7)	Ti1—C6—H6	120.9
C8—Ti1—C2	82.67 (8)	C6—C7—C8	107.3 (2)
Cl1—Ti1—C2	136.63 (5)	C6—C7—Ti1	75.40 (12)
C7—Ti1—C2	96.71 (8)	C8—C7—Ti1	71.89 (12)
Cl2—Ti1—C2	92.88 (5)	C6—C7—H7	126.3
C5—Ti1—C2	58.37 (7)	C8—C7—H7	126.3
C1—Ti1—C2	34.40 (7)	Ti1—C7—H7	118.4
C4—Ti1—C2	57.42 (7)	C9—C8—C7	107.6 (2)
C8—Ti1—C3	116.06 (8)	C9—C8—Ti1	75.41 (12)
Cl1—Ti1—C3	106.85 (5)	C7—C8—Ti1	73.07 (12)
C7—Ti1—C3	129.31 (8)	C9—C8—H8	126.2
Cl2—Ti1—C3	76.78 (5)	C7—C8—H8	126.2
C5—Ti1—C3	57.17 (7)	Ti1—C8—H8	117.4
C1—Ti1—C3	56.08 (7)	C8—C9—C10	109.6 (2)
C4—Ti1—C3	33.64 (7)	C8—C9—Ti1	70.32 (12)
C2—Ti1—C3	34.57 (7)	C10—C9—Ti1	75.35 (12)
C8—Ti1—C9	34.27 (8)	C8—C9—H9	125.2
Cl1—Ti1—C9	117.28 (6)	C10—C9—H9	125.2
C7—Ti1—C9	56.96 (8)	Ti1—C9—H9	120.8
Cl2—Ti1—C9	78.16 (6)	C9—C10—C6	105.27 (19)
C5—Ti1—C9	143.12 (7)	C9—C10—Si3	127.46 (17)
C1—Ti1—C9	113.16 (7)	C6—C10—Si3	125.99 (17)
C4—Ti1—C9	162.98 (8)	C9—C10—Ti1	70.91 (12)
C2—Ti1—C9	106.05 (8)	C6—C10—Ti1	71.21 (12)
C3—Ti1—C9	130.21 (8)	Si3—C10—Ti1	132.15 (10)
C8—Ti1—C6	56.67 (8)	Si3—C11—H11A	109.5
Cl1—Ti1—C6	79.64 (6)	Si3—C11—H11B	109.5
C7—Ti1—C6	33.81 (8)	H11A—C11—H11B	109.5
Cl2—Ti1—C6	121.39 (6)	Si3—C11—H11C	109.5
C5—Ti1—C6	106.42 (7)	H11A—C11—H11C	109.5
C1—Ti1—C6	104.86 (7)	H11B—C11—H11C	109.5
C4—Ti1—C6	136.68 (7)	Si3—C12—H12A	109.5
C2—Ti1—C6	130.51 (7)	Si3—C12—H12B	109.5
C3—Ti1—C6	160.80 (7)	H12A—C12—H12B	109.5
C9—Ti1—C6	55.71 (8)	Si3—C12—H12C	109.5
C8—Ti1—C10	57.12 (8)	H12A—C12—H12C	109.5
Cl1—Ti1—C10	84.58 (5)	H12B—C12—H12C	109.5
C7—Ti1—C10	57.03 (7)	Si3—C13—H13A	109.5
Cl2—Ti1—C10	87.78 (5)	Si3—C13—H13B	109.5
C5—Ti1—C10	140.22 (7)	H13A—C13—H13B	109.5
C1—Ti1—C10	131.20 (7)	Si3—C13—H13C	109.5
C4—Ti1—C10	163.27 (7)	H13A—C13—H13C	109.5
C2—Ti1—C10	138.47 (7)	H13B—C13—H13C	109.5
C3—Ti1—C10	161.24 (7)	Si1—C14—H14A	109.5
C9—Ti1—C10	33.74 (7)	Si1—C14—H14B	109.5
C6—Ti1—C10	33.82 (7)	H14A—C14—H14B	109.5
C16—Si1—C15	108.75 (19)	Si1—C14—H14C	109.5
C16—Si1—C14	110.49 (16)	H14A—C14—H14C	109.5
C15—Si1—C14	110.56 (14)	H14B—C14—H14C	109.5
C16—Si1—C2	104.80 (12)	Si1—C15—H15A	109.5
C15—Si1—C2	111.50 (11)	Si1—C15—H15B	109.5
C14—Si1—C2	110.59 (10)	H15A—C15—H15B	109.5
C19—Si2—C18	110.54 (16)	Si1—C15—H15C	109.5
C19—Si2—C17	110.16 (15)	H15A—C15—H15C	109.5
C18—Si2—C17	109.88 (13)	H15B—C15—H15C	109.5
C19—Si2—C5	107.49 (12)	Si1—C16—H16A	109.5
C18—Si2—C5	112.99 (11)	Si1—C16—H16B	109.5
C17—Si2—C5	105.65 (11)	H16A—C16—H16B	109.5
C12—Si3—C11	109.97 (17)	Si1—C16—H16C	109.5
C12—Si3—C13	109.98 (17)	H16A—C16—H16C	109.5
C11—Si3—C13	109.33 (15)	H16B—C16—H16C	109.5
C12—Si3—C10	111.98 (12)	Si2—C17—H17A	109.5
C11—Si3—C10	105.61 (12)	Si2—C17—H17B	109.5
C13—Si3—C10	109.86 (12)	H17A—C17—H17B	109.5
C2—C1—C5	111.03 (17)	Si2—C17—H17C	109.5
C2—C1—Ti1	73.01 (12)	H17A—C17—H17C	109.5
C5—C1—Ti1	72.75 (12)	H17B—C17—H17C	109.5
C2—C1—H1	124.5	Si2—C18—H18A	109.5
C5—C1—H1	124.5	Si2—C18—H18B	109.5
Ti1—C1—H1	121.3	H18A—C18—H18B	109.5
C1—C2—C3	104.91 (17)	Si2—C18—H18C	109.5
C1—C2—Si1	129.21 (15)	H18A—C18—H18C	109.5
C3—C2—Si1	124.29 (15)	H18B—C18—H18C	109.5
C1—C2—Ti1	72.59 (11)	Si2—C19—H19A	109.5
C3—C2—Ti1	72.86 (12)	Si2—C19—H19B	109.5
Si1—C2—Ti1	130.06 (11)	H19A—C19—H19B	109.5
C4—C3—C2	109.76 (18)	Si2—C19—H19C	109.5
C4—C3—Ti1	72.84 (12)	H19A—C19—H19C	109.5
C2—C3—Ti1	72.57 (12)	H19B—C19—H19C	109.5
			
C8—Ti1—C1—C2	−92.10 (13)	C10—Ti1—C5—C4	153.45 (12)
Cl1—Ti1—C1—C2	130.24 (10)	C8—Ti1—C5—Si2	92.64 (13)
C7—Ti1—C1—C2	−127.73 (13)	Cl1—Ti1—C5—Si2	−45.01 (12)
Cl2—Ti1—C1—C2	7.78 (14)	C7—Ti1—C5—Si2	62.81 (13)
C5—Ti1—C1—C2	119.20 (17)	Cl2—Ti1—C5—Si2	−136.90 (9)
C4—Ti1—C1—C2	79.99 (13)	C1—Ti1—C5—Si2	125.39 (19)
C3—Ti1—C1—C2	39.25 (11)	C4—Ti1—C5—Si2	−122.03 (19)
C9—Ti1—C1—C2	−84.70 (13)	C2—Ti1—C5—Si2	160.79 (16)
C6—Ti1—C1—C2	−143.33 (12)	C3—Ti1—C5—Si2	−158.12 (16)
C10—Ti1—C1—C2	−118.84 (13)	C9—Ti1—C5—Si2	87.04 (17)
C8—Ti1—C1—C5	148.70 (13)	C6—Ti1—C5—Si2	32.91 (14)
Cl1—Ti1—C1—C5	11.05 (13)	C10—Ti1—C5—Si2	31.42 (19)
C7—Ti1—C1—C5	113.08 (13)	C8—Ti1—C6—C7	−38.83 (14)
Cl2—Ti1—C1—C5	−111.42 (11)	Cl1—Ti1—C6—C7	146.12 (14)
C4—Ti1—C1—C5	−39.20 (11)	Cl2—Ti1—C6—C7	−125.34 (13)
C2—Ti1—C1—C5	−119.20 (17)	C5—Ti1—C6—C7	63.59 (15)
C3—Ti1—C1—C5	−79.94 (13)	C1—Ti1—C6—C7	27.80 (15)
C9—Ti1—C1—C5	156.11 (12)	C4—Ti1—C6—C7	84.20 (17)
C6—Ti1—C1—C5	97.47 (12)	C2—Ti1—C6—C7	1.45 (18)
C10—Ti1—C1—C5	121.96 (12)	C3—Ti1—C6—C7	34.3 (3)
C5—C1—C2—C3	−3.0 (2)	C9—Ti1—C6—C7	−80.35 (15)
Ti1—C1—C2—C3	−66.23 (14)	C10—Ti1—C6—C7	−118.1 (2)
C5—C1—C2—Si1	−168.80 (16)	C8—Ti1—C6—C10	79.29 (14)
Ti1—C1—C2—Si1	127.93 (18)	Cl1—Ti1—C6—C10	−95.75 (12)
C5—C1—C2—Ti1	63.27 (15)	C7—Ti1—C6—C10	118.1 (2)
C16—Si1—C2—C1	107.6 (2)	Cl2—Ti1—C6—C10	−7.22 (15)
C15—Si1—C2—C1	−134.9 (2)	C5—Ti1—C6—C10	−178.29 (12)
C14—Si1—C2—C1	−11.4 (2)	C1—Ti1—C6—C10	145.92 (13)
C16—Si1—C2—C3	−55.7 (2)	C4—Ti1—C6—C10	−157.68 (12)
C15—Si1—C2—C3	61.7 (2)	C2—Ti1—C6—C10	119.58 (13)
C14—Si1—C2—C3	−174.82 (19)	C3—Ti1—C6—C10	152.5 (2)
C16—Si1—C2—Ti1	−151.92 (18)	C9—Ti1—C6—C10	37.77 (13)
C15—Si1—C2—Ti1	−34.44 (19)	C10—C6—C7—C8	0.2 (2)
C14—Si1—C2—Ti1	89.01 (16)	Ti1—C6—C7—C8	65.30 (14)
C8—Ti1—C2—C1	82.40 (13)	C10—C6—C7—Ti1	−65.13 (15)
Cl1—Ti1—C2—C1	−74.67 (13)	C8—Ti1—C7—C6	114.2 (2)
C7—Ti1—C2—C1	50.21 (13)	Cl1—Ti1—C7—C6	−35.16 (15)
Cl2—Ti1—C2—C1	−173.79 (11)	Cl2—Ti1—C7—C6	80.19 (16)
C5—Ti1—C2—C1	−35.46 (11)	C5—Ti1—C7—C6	−120.78 (14)
C4—Ti1—C2—C1	−76.73 (13)	C1—Ti1—C7—C6	−152.15 (15)
C3—Ti1—C2—C1	−112.26 (17)	C4—Ti1—C7—C6	−124.12 (14)
C9—Ti1—C2—C1	107.71 (12)	C2—Ti1—C7—C6	−178.89 (14)
C6—Ti1—C2—C1	49.40 (15)	C3—Ti1—C7—C6	−166.13 (13)
C10—Ti1—C2—C1	96.29 (14)	C9—Ti1—C7—C6	76.30 (15)
C8—Ti1—C2—C3	−165.34 (13)	C10—Ti1—C7—C6	35.82 (13)
Cl1—Ti1—C2—C3	37.60 (15)	Cl1—Ti1—C7—C8	−149.32 (12)
C7—Ti1—C2—C3	162.47 (13)	Cl2—Ti1—C7—C8	−33.97 (17)
Cl2—Ti1—C2—C3	−61.53 (12)	C5—Ti1—C7—C8	125.06 (14)
C5—Ti1—C2—C3	76.81 (13)	C1—Ti1—C7—C8	93.69 (14)
C1—Ti1—C2—C3	112.26 (17)	C4—Ti1—C7—C8	121.71 (14)
C4—Ti1—C2—C3	35.53 (12)	C2—Ti1—C7—C8	66.95 (14)
C9—Ti1—C2—C3	−140.03 (12)	C3—Ti1—C7—C8	79.71 (16)
C6—Ti1—C2—C3	161.66 (12)	C9—Ti1—C7—C8	−37.86 (13)
C10—Ti1—C2—C3	−151.44 (12)	C6—Ti1—C7—C8	−114.2 (2)
C8—Ti1—C2—Si1	−44.60 (13)	C10—Ti1—C7—C8	−78.35 (14)
Cl1—Ti1—C2—Si1	158.33 (8)	C6—C7—C8—C9	0.4 (2)
C7—Ti1—C2—Si1	−76.79 (14)	Ti1—C7—C8—C9	68.09 (14)
Cl2—Ti1—C2—Si1	59.20 (12)	C6—C7—C8—Ti1	−67.66 (15)
C5—Ti1—C2—Si1	−162.46 (16)	Cl1—Ti1—C8—C9	−69.53 (16)
C1—Ti1—C2—Si1	−127.00 (19)	C7—Ti1—C8—C9	−113.98 (19)
C4—Ti1—C2—Si1	156.26 (16)	Cl2—Ti1—C8—C9	42.00 (14)
C3—Ti1—C2—Si1	120.73 (19)	C5—Ti1—C8—C9	−174.02 (13)
C9—Ti1—C2—Si1	−19.30 (15)	C1—Ti1—C8—C9	167.85 (15)
C6—Ti1—C2—Si1	−77.61 (16)	C4—Ti1—C8—C9	158.46 (13)
C10—Ti1—C2—Si1	−30.71 (19)	C2—Ti1—C8—C9	133.15 (14)
C1—C2—C3—C4	2.2 (2)	C3—Ti1—C8—C9	123.95 (13)
Si1—C2—C3—C4	168.97 (16)	C6—Ti1—C8—C9	−76.56 (14)
Ti1—C2—C3—C4	−63.81 (15)	C10—Ti1—C8—C9	−35.92 (13)
C1—C2—C3—Ti1	66.04 (14)	Cl1—Ti1—C8—C7	44.45 (17)
Si1—C2—C3—Ti1	−127.22 (16)	Cl2—Ti1—C8—C7	155.98 (12)
C8—Ti1—C3—C4	134.12 (13)	C5—Ti1—C8—C7	−60.04 (14)
Cl1—Ti1—C3—C4	−36.15 (12)	C1—Ti1—C8—C7	−78.17 (14)
C7—Ti1—C3—C4	95.15 (14)	C4—Ti1—C8—C7	−87.56 (16)
Cl2—Ti1—C3—C4	−126.51 (12)	C2—Ti1—C8—C7	−112.87 (14)
C5—Ti1—C3—C4	37.31 (12)	C3—Ti1—C8—C7	−122.07 (13)
C1—Ti1—C3—C4	78.83 (13)	C9—Ti1—C8—C7	113.98 (19)
C2—Ti1—C3—C4	117.89 (18)	C6—Ti1—C8—C7	37.42 (13)
C9—Ti1—C3—C4	171.82 (12)	C10—Ti1—C8—C7	78.06 (14)
C6—Ti1—C3—C4	71.2 (3)	C7—C8—C9—C10	−0.9 (2)
C10—Ti1—C3—C4	−161.9 (2)	Ti1—C8—C9—C10	65.64 (15)
C8—Ti1—C3—C2	16.23 (15)	C7—C8—C9—Ti1	−66.51 (14)
Cl1—Ti1—C3—C2	−154.04 (11)	Cl1—Ti1—C9—C8	132.99 (12)
C7—Ti1—C3—C2	−22.74 (16)	C7—Ti1—C9—C8	38.74 (13)
Cl2—Ti1—C3—C2	115.60 (12)	Cl2—Ti1—C9—C8	−138.45 (14)
C5—Ti1—C3—C2	−80.58 (13)	C5—Ti1—C9—C8	9.4 (2)
C1—Ti1—C3—C2	−39.06 (12)	C1—Ti1—C9—C8	−13.02 (16)
C4—Ti1—C3—C2	−117.89 (18)	C4—Ti1—C9—C8	−61.7 (3)
C9—Ti1—C3—C2	53.93 (15)	C2—Ti1—C9—C8	−48.84 (15)
C6—Ti1—C3—C2	−46.7 (3)	C3—Ti1—C9—C8	−77.35 (16)
C10—Ti1—C3—C2	80.2 (3)	C6—Ti1—C9—C8	79.62 (14)
C2—C3—C4—C5	−0.7 (2)	C10—Ti1—C9—C8	117.49 (19)
Ti1—C3—C4—C5	−64.39 (15)	C8—Ti1—C9—C10	−117.49 (19)
C2—C3—C4—Ti1	63.64 (15)	Cl1—Ti1—C9—C10	15.50 (14)
C8—Ti1—C4—C3	−66.74 (16)	C7—Ti1—C9—C10	−78.75 (14)
Cl1—Ti1—C4—C3	144.92 (12)	Cl2—Ti1—C9—C10	104.06 (12)
C7—Ti1—C4—C3	−110.83 (13)	C5—Ti1—C9—C10	−108.06 (15)
Cl2—Ti1—C4—C3	52.11 (12)	C1—Ti1—C9—C10	−130.51 (12)
C5—Ti1—C4—C3	−116.70 (17)	C4—Ti1—C9—C10	−179.2 (2)
C1—Ti1—C4—C3	−77.84 (13)	C2—Ti1—C9—C10	−166.34 (12)
C2—Ti1—C4—C3	−36.52 (12)	C3—Ti1—C9—C10	165.16 (12)
C9—Ti1—C4—C3	−21.8 (3)	C6—Ti1—C9—C10	−37.87 (12)
C6—Ti1—C4—C3	−153.01 (13)	C8—C9—C10—C6	0.9 (2)
C10—Ti1—C4—C3	159.7 (2)	Ti1—C9—C10—C6	63.40 (14)
C8—Ti1—C4—C5	49.96 (17)	C8—C9—C10—Si3	168.54 (15)
Cl1—Ti1—C4—C5	−98.38 (11)	Ti1—C9—C10—Si3	−129.01 (16)
C7—Ti1—C4—C5	5.87 (16)	C8—C9—C10—Ti1	−62.45 (15)
Cl2—Ti1—C4—C5	168.81 (11)	C7—C6—C10—C9	−0.7 (2)
C1—Ti1—C4—C5	38.85 (11)	Ti1—C6—C10—C9	−63.20 (14)
C2—Ti1—C4—C5	80.18 (13)	C7—C6—C10—Si3	−168.51 (15)
C3—Ti1—C4—C5	116.70 (17)	Ti1—C6—C10—Si3	128.97 (16)
C9—Ti1—C4—C5	94.9 (3)	C7—C6—C10—Ti1	62.52 (15)
C6—Ti1—C4—C5	−36.31 (17)	C12—Si3—C10—C9	55.8 (2)
C10—Ti1—C4—C5	−83.6 (3)	C11—Si3—C10—C9	−63.8 (2)
C2—C1—C5—C4	2.5 (2)	C13—Si3—C10—C9	178.4 (2)
Ti1—C1—C5—C4	65.97 (14)	C12—Si3—C10—C6	−139.0 (2)
C2—C1—C5—Si2	170.91 (16)	C11—Si3—C10—C6	101.3 (2)
Ti1—C1—C5—Si2	−125.65 (17)	C13—Si3—C10—C6	−16.5 (2)
C2—C1—C5—Ti1	−63.44 (15)	C12—Si3—C10—Ti1	−42.1 (2)
C3—C4—C5—C1	−1.1 (2)	C11—Si3—C10—Ti1	−161.77 (16)
Ti1—C4—C5—C1	−66.06 (14)	C13—Si3—C10—Ti1	80.43 (18)
C3—C4—C5—Si2	−169.83 (16)	C8—Ti1—C10—C9	36.50 (13)
Ti1—C4—C5—Si2	125.18 (16)	Cl1—Ti1—C10—C9	−166.20 (12)
C3—C4—C5—Ti1	64.99 (15)	C7—Ti1—C10—C9	78.53 (14)
C19—Si2—C5—C1	−167.0 (2)	Cl2—Ti1—C10—C9	−71.83 (12)
C18—Si2—C5—C1	70.8 (2)	C5—Ti1—C10—C9	116.91 (14)
C17—Si2—C5—C1	−49.4 (2)	C1—Ti1—C10—C9	68.30 (15)
C19—Si2—C5—C4	−0.8 (2)	C4—Ti1—C10—C9	179.2 (2)
C18—Si2—C5—C4	−123.0 (2)	C2—Ti1—C10—C9	20.02 (17)
C17—Si2—C5—C4	116.8 (2)	C3—Ti1—C10—C9	−37.4 (3)
C19—Si2—C5—Ti1	94.86 (17)	C6—Ti1—C10—C9	114.34 (18)
C18—Si2—C5—Ti1	−27.36 (17)	C8—Ti1—C10—C6	−77.84 (14)
C17—Si2—C5—Ti1	−147.53 (14)	Cl1—Ti1—C10—C6	79.46 (13)
C8—Ti1—C5—C1	−32.75 (13)	C7—Ti1—C10—C6	−35.80 (13)
Cl1—Ti1—C5—C1	−170.40 (11)	Cl2—Ti1—C10—C6	173.84 (13)
C7—Ti1—C5—C1	−62.58 (12)	C5—Ti1—C10—C6	2.57 (19)
Cl2—Ti1—C5—C1	97.70 (12)	C1—Ti1—C10—C6	−46.04 (16)
C4—Ti1—C5—C1	112.58 (17)	C4—Ti1—C10—C6	64.9 (3)
C2—Ti1—C5—C1	35.39 (11)	C2—Ti1—C10—C6	−94.31 (15)
C3—Ti1—C5—C1	76.49 (12)	C3—Ti1—C10—C6	−151.8 (2)
C9—Ti1—C5—C1	−38.36 (18)	C9—Ti1—C10—C6	−114.34 (18)
C6—Ti1—C5—C1	−92.48 (12)	C8—Ti1—C10—Si3	160.20 (18)
C10—Ti1—C5—C1	−93.97 (15)	Cl1—Ti1—C10—Si3	−42.50 (14)
C8—Ti1—C5—C4	−145.33 (12)	C7—Ti1—C10—Si3	−157.76 (18)
Cl1—Ti1—C5—C4	77.02 (11)	Cl2—Ti1—C10—Si3	51.88 (14)
C7—Ti1—C5—C4	−175.16 (13)	C5—Ti1—C10—Si3	−119.39 (14)
Cl2—Ti1—C5—C4	−14.87 (14)	C1—Ti1—C10—Si3	−168.00 (12)
C1—Ti1—C5—C4	−112.58 (17)	C4—Ti1—C10—Si3	−57.1 (3)
C2—Ti1—C5—C4	−77.18 (12)	C2—Ti1—C10—Si3	143.73 (13)
C3—Ti1—C5—C4	−36.09 (12)	C3—Ti1—C10—Si3	86.3 (3)
C9—Ti1—C5—C4	−150.93 (14)	C9—Ti1—C10—Si3	123.7 (2)
C6—Ti1—C5—C4	154.94 (12)	C6—Ti1—C10—Si3	−122.0 (2)
